# Determinants of body mass index during early life: findings from an exposome-wide association study with follow-up replication and Mendelian randomization analyses

**DOI:** 10.1093/exposome/osaf004

**Published:** 2025-05-02

**Authors:** Jie V. Zhao, Ana Goncalves Soares, Demetris Avraam, Tim Cadman, Marie Aline Charles, Barbara Heude, Vincent Jaddoe, Yannis Manios, George Moschonis, Anne-Marie Nybo Andersen, Lorenzo Richiardi, Justiina Ronkainen, Sylvain Sebert, Morris Swertz, Evangelia Tzorovili, Martine Vrijheid, Wen Lun Yuan, Deborah A. Lawlor, Ahmed Elhakeem

**Affiliations:** 1 School of Public Health, The University of Hong Kong, Hong Kong, China; 2 MRC Integrative Epidemiology Unit, University of Bristol, Bristol, UK; 3 Population Health Science, Bristol Medical School, University of Bristol, UK; 4 Section of Epidemiology, Department of Public Health, University of Copenhagen, Denmark; 5 ISGlobal, Barcelona, Spain; 6 Genetics Department (GCC—Genomic Coordination Centre), University Medical Centre Groningen, Groningen, The Netherlands; 7 Joint Unit Elfe, Ined, Inserm, EFS, Aubervilliers, France; 8 Université Paris Cité and Université Sorbonne Paris Nord, Inserm, INRAE, Center for Research in Epidemiology and StatisticS (CRESS), Paris, F-75004, France; 9 Department of Pediatrics, Erasmus Medical Center, University Medical Center, The Generation R Study Group, (Na 29-18), PO Box 2040, 3000 CA Rotterdam, The Netherland; 10 Generation R Study Group, Erasmus Medical Center, University Medical Center, PO Box 2040, 3000 CA Rotterdam, The Netherlands; 11 Department of Nutrition and Dietetics, School of Health Science and Education, Harokopio University, Athens 17671, Greece; 12 Institute of Agri-Food and Life Sciences, Hellenic Mediterranean University Research Center, Heraklion 71410, Greece; 13 Department of Food, Nutrition and Dietetics, School of Allied Health, Human Services and Sport, La Trobe University, Australia; 14 La Trobe Institute for Sustainable Agriculture & Food (LISAF), La Trobe University, VIC 3086, Australia; 15 Cancer Epidemiology Unit, Department of Medical Sciences, University of Turin, Reference Centre for Epidemiology and Cancer Prevention Piemonte, Turin, Italy; 16 Research Unit of Population Health, Faculty of Medicine, University of Oulu, Oulu, Finland; 17 Department of Nutrition and Dietetics, School of Health Science and Education, Harokopio University, Athens 17671, Greece; 18 Universitat Pompeu Fabra (UPF), Barcelona, Spain; 19 CIBER Epidemiologíay Salud Pública (CIBERESP), Spain

**Keywords:** exposome-wide association study, body mass index, ALSPAC, replication, Mendelian randomization, early life

## Abstract

**Background:**

Studies of factors influencing body mass index (BMI) mostly focus on hypothesized/established factors. We aimed to explore potential factors influencing BMI across childhood and adolescence using a hypothesis-free exposome-wide association study (ExWAS), with external replication and Mendelian randomization (MR).

**Methods:**

The ExWAS was done in the Avon Longitudinal Study of Parents and Children (ALSPAC) using univariable regressions to estimate prospective associations of 2,523 exposures with BMI in infancy (up to 12 months, *N =* 12 761), early-childhood (13–60 months, *N =* 12 138), middle-childhood (61 months to 12 years, *N =* 9,105) and adolescence (13–18 years, *N =* 7379). Associations passing Bonferroni correction were followed-up with confounder-adjusted regression in ALSPAC. Replication of confounder-adjusted associations were explored in eight independent cohorts and/or MR analyses in ALSPAC.

**Results:**

The ExWAS identified 8, 16, 66 and 82 exposures prospectively associated with BMI in infancy, early-childhood, middle-childhood, and adolescence, respectively. Of these, 8, 11, 42, and 26 remained associated following confounder adjustment. Replicated associations included maternal BMI, smoking and fat intake; only maternal smoking was supported by MR. Associations of more TV viewing time with higher BMI at one or more ages was supported by MR showing more time spent watching TV in early-childhood leads to higher BMI in middle-childhood. It was not possible to explore replication or MR for confounder-adjusted associations of offspring emotion, sociability, and peer problems with subsequent BMI.

**Conclusions:**

Our ExWAS suggests more TV viewing time in early-childhood increases BMI in mid-childhood. Novel potential effects of social, emotional and peer exposures in childhood need replication and assessment of causality.

## Background

Higher body mass index (BMI) in childhood is associated with several adverse outcomes, including poor mental health and cardiometabolic risk factors.^1,2^ It tracks into adulthood,^3^ with recent Mendelian randomization (MR) analyses suggesting higher mean childhood BMI increases adult cardiovascular diseases, through its relationship with adult higher mean BMI.^2^ Most conventional multivariable regression and MR analyses to date have explored relationships of single or a small number of hypothesized risk factors for variation in childhood BMI,^4–7^ such as maternal smoking,^8^ and children’s behavior and nutritional factors, such as unhealthy diet and physical inactivity.^6,9^ This can result in a literature overload in one area and failure to identify potential novel risk factors.

Exposome-wide association studies (ExWAS) are a hypothesis free approach that aims to use the totality of non-genetic life-course exposures to identify potential novel risk factors for an outcome of interest.^10–12^ To date, there have been three ExWAS exploring potential effects of exposures on variation in childhood BMI.^13–15^ The sample sizes in these ExWAS ranged from 1224 to 3,618 and included between 139 and 441 exposures, and included different categories of exposures from each other. Two of them were cross-sectional or partly cross-sectional with exposures and childhood BMI measured at the same time.^13,14^ None of these studies attempted to explore replication or were unable to conduct replication in independent studies which is essential in hypothesis free studies.^16^

Potential risk factors for variation in childhood BMI or their associations with BMI may change through childhood and into adolescence, and identifying different potential risk factors for higher mean BMI at different ages might provide important insights into age-specific prevention, but the previous ExWAS did not explore potential effects of multiple exposures on variation in childhood BMI at different ages across childhood.

The aim of this study was to explore the potential effects of multiple early life exposures on variation in BMI from infancy to adolescence. We addressed the limitations of previous ExWAS of childhood BMI, by undertaking a prospective unadjusted linear regression ExWAS of 2523 exposures with variation in BMI in infancy (*N* = 12 761), early- (*N* = 12 138), middle-childhood (*N* = 9105) and adolescence (*N* = 7379). This was followed by prior defined confounder adjustment in the same cohort for studies that reached our predefined multiple testing threshold, and external replication and/or MR of associations that remained after confounder adjustment.

## Methods

### Study design summary

**Figure 1:**
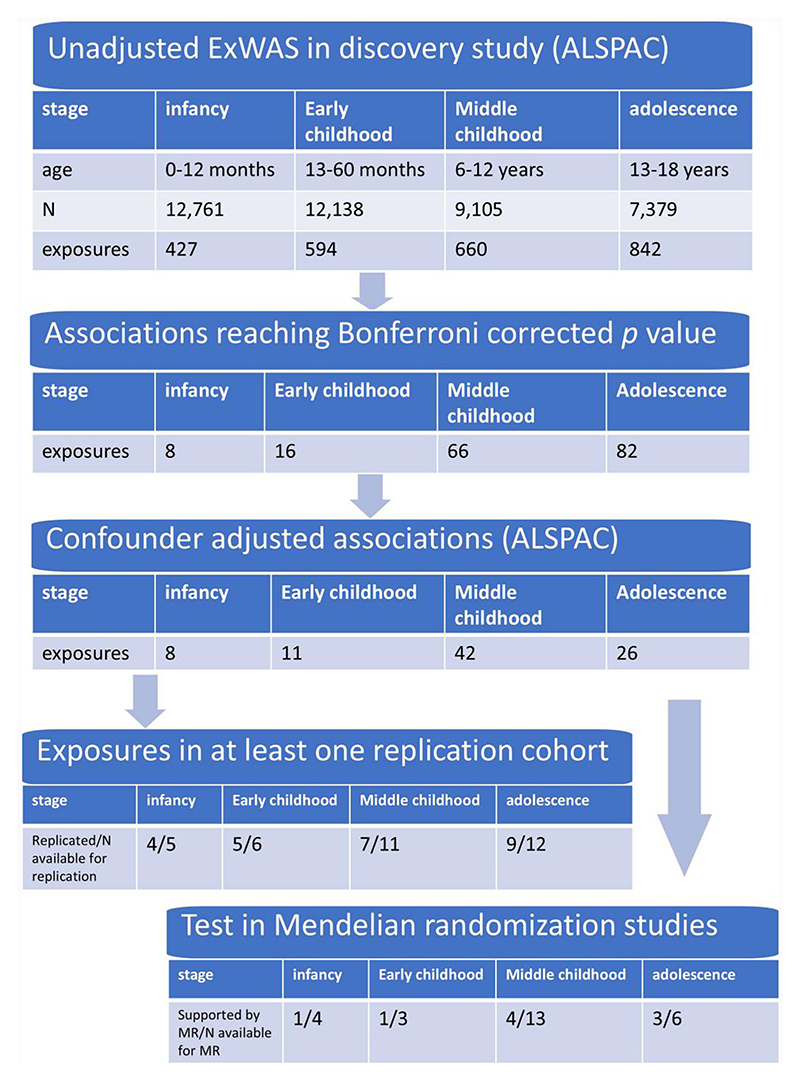
Flow chart of the study design.

Figure 1 summarizes all stages of the study design. We used the Avon Longitudinal Study of Parents and Children (ALSPAC)^17,18^ as the discovery cohort, and explored potential effects of a wide range of maternal, family, environment, and child exposures, including air pollution, area-level deprivation index, individual level socioeconomic position, health, and behaviors such as smoking, detailed dietary factors and physical activity. Only prospective associations were explored (ie, with exposure assessed before the timing of the BMI measure). In total 2523 exposure outcome associations were included in the study with numbers being smallest (427) in relation to BMI in the first 12 months of life and highest in the 13-18 years old analyses (842). We conducted unadjusted analysis and selected exposures passing Bonferroni-corrected statistical significance to be taken forward for confounder adjusted multivariable regression in ALSPAC. For the exposures that remained after confounder adjustment, we sought replication in pooled analyses from eight independent European cohorts and/or MR evidence. Given the hypothesis free approach of this study and the likelihood that there would be few or no genome-wide analyses of many of the exposures assessed in childhood or of BMI in the age groups used here, we *a priori* decided to review the literature for any relevant published MR studies and to undertake one-sample MR where possible in ALSPAC.

### Participating cohorts

Details of exposure and confounder selection and of how these were assessed in the cohorts are described in relevant sections below. Here we described the place, timing and recruitment of cohort participants.

#### Discovery cohort

ALSPAC is a prospective birth cohort study that recruited pregnant women residing in the former county of Avon, South West England, with an expected date of delivery between April 1991 and December 1992.^17–19^ In total, 14541 women were enrolled into ALSPAC, with 14901 children born. The participants have been followed up since birth, with questionnaires, research clinics and linkage to routine health and education data.^20^ All participants have given informed consent to be involved in the ALSPAC study. Ethical approval was obtained from the ALSPAC Ethics and Law Committee and Local Research Ethics. The ALSPAC website contains details of all the data that is available through a fully searchable data dictionary and variable search tool (http://www.bristol.ac.uk/alspac/researchers/our-data/). We explored associations with BMI in four age groups using the extensive repeat measurements of weight and height in ALSPAC: up to 12 months (infancy, *N* =12 761), 13-60 months (early-childhood, *N* = 12 138), 61 months to 12 years (middle-childhood, *N* = 9105) and 13-18 years (adolescence, *N* = 7379) (Supplemental Table 1).

#### Replication cohorts

Replication was explored in cohorts of European ancestry from the EU Child Cohort Network.^21,22^ Eight independent cohorts in the Network, including up to 75212 participants, met our prior defied criteria: (1) had data on at least one of the relevant exposures and BMI, and (2) had data on all the confounders used in the discovery cohort. These cohorts were Nascita e INFanzia: gli Effetti dell’Ambiente (NINFEA), Danish National Birth Cohort (DNBC), Generation R Study (GenR), Etude Longitudinale Française depuis l’Enfance (ELFE), Etude des déterminants pré et postnatals du développement de la santé de l’enfant (EDEN), Northern Finland Birth Cohorts 1966 and 1986 *(*NFBC1966, NFBC1986), and Healthy Growth Study (HGS). More detailed descriptions for each replication cohort can be found in the Supplemental Methods. A summary of the age group(s) used, number of participants, mean and standard deviation (SD) of BMI at the age group(s) for each of these cohorts is shown in Supplemental Table 2.

### Discovery ExWAS

#### Assessment of child BMI

Numerous repeat weight and height measures have been obtained from various sources from birth to adulthood (mid-20s) in ALSPAC, including from routinely collected child health records (from birth to 5 years), parent report collected from questionnaires, and ALSPAC research clinic assessments.^23–25^ We only used data obtained from ALSPAC research clinics and health records, to minimize bias due to measurement error. We prioritized the use of ALSPAC clinic data. Where clinic data was not available in any participant in any age strata we used data extracted from health records.^25^ Where participants had more than one measure of weight and height within an age stratum we prioritized measurements taken at the oldest age. Weight was measured to the nearest 50 g using a Tanita Body Fat Analyser, and height the nearest 0.1 cm using a Harpenden stadiometer; for both measures participants were unshod and in light clothing. Supplemental Table 1 summarizes the source of measurement data and median, interquartile range (IQR) and full range of age of measurement within each age strata.

#### Maternal and child exposures

We *a priori* defined exposures that we would include in the ExWAS and any potential factor that could influence variation in BMI that was assessed prior to BMI in a given age group. Limiting to potential factors that could influence variation in BMI means that we exclude technical data, such as the type of equipment or questionnaire used to obtain data. By only including exposures that were measured before BMI at each given age, all analyses are prospective. This meant for example family level (eg, parental socioeconomic position) and maternal (intrauterine) exposures were explored in relation to BMI in all four age groups. This definition also means we explored (previous) childhood BMI at one time point as exposures for outcome BMI measured at a later age. Supplemental Table 3 provides complete lists of all exposures by age strata. We selected all exposures measured prior to the lowest age of BMI measurement within each age strata. The selected variables include a range of types of exposures, including environmental (eg, several types of pollution, proximity to green space, traffic, area level and built environment), family socioeconomic (eg, residential area deprivation, maternal education, income), behavioral (eg, parental and/or child smoking, diet, physical activities) and include exposures across the life course up relevant to each strata.

#### Confounder selection and assessment

We adjusted for the following confounders for all exposures: maternal age, education, parity, BMI, smoking pre-pregnancy and during pregnancy, and ethnicity (apart from when these were the exposures of interest), and childhood prior BMI (only included as a confounder for children’s exposures occurring after the specific BMI measure, and hence not for any maternal exposures). The detailed assessment method for each confounder is shown in Supplemental Methods. We a-priori did not consider children’s age and sex at the time of BMI assessment to be confounders, as we did not consider that they would cause variation in the maternal and child exposures. However, as they are strongly associated with childhood BMI and adjusting for them could improve the statistical efficiency of the associations. For that reason we undertook additional analyses adjusting for sex and age at the time of BMI assessment in addition to our pre-specified confounders.

### Statistical analysis

We used linear regression to estimate the unadjusted and confounder-adjusted associations of each exposure with BMI. For unadjusted analyses, we used a Bonferroni corrected *P*-value threshold, which took into account all association tests across all age groups, with the threshold being 1.98*10^−5^ (ie, 0.05 divided by the number of tests 2523), to select which of the unadjusted associations would be taken forward for confounder adjusted analyses. We compared the adjusted with unadjusted associations in ALSPAC and defined those that attenuated by more than 50% as possibly being explained by confounding. This approach did not rely on the conventional *P*-value of 0.05 and took account of differences in statistical power between models of different sample sizes and covariables.

### Replication in independent cohorts

For every cohort that was able to contribute to a specific exposure age period analysis (ie, had complete data on the specific exposure, BMI in the age category and all confounders) (data-catalogue.molgeniscloud.org/catalogue), multivariable linear regression was undertaken in each cohort using the same adjustments as in the discovery ExWAS. The measures for exposure, outcome and confounders have been harmonized in different cohorts. Results were then pooled using random-effects meta-analysis. The analysis in each cohort was undertaken using the DataSHIELD platform (https://www.datashield.org/),^26^ which supports federated analyses (ie, individual participant data remained on cohort-specific servers^27^). We took into account of the sample size differences between the adjusted results in ALSPAC and the replication cohorts (Supplemental Table 4), and defined results as replicated with the following criteria:

- Pooled replication sample size equal to or smaller than ALSPAC: results have consistent directions to results in ALSPAC, and the results among the replication cohorts have similar magnitude based on heterogeneity having I^2^ <50%; or
- Pooled replication sample size larger than ALSPAC: results have consistent directions to results in ALSPAC and p-value in the pooled replication cohorts ≤0.05.

### MR

For all the confounder-adjusted exposure-BMI associations in any age category that were taken forward into follow-up (Figure 1), we explored the possibility of obtaining relevant MR results. As most MR studies to date have been based on genome-wide association studies (GWAS) in adults, we did not anticipate many published GWAS of the exposures in earlier ages.^28,29^ Therefore, we decided to take two approaches to explore MR evidence for follow-up associations.

First, we searched the literature for any published (one- or two-sample) MR studies on the potential effects of follow-up exposures on childhood BMI (See Supplemental Table 5 for search terms used for each follow-up exposure). In this search we included studies of childhood BMI of any age up to 18 years, with the idea that we would then consider their relevance to the specific age group. Second, we explored the potential of undertaking one-sample MR in ALSPAC, where we could identify GWAS of any of the relevant exposures. Where such a GWAS was identified we generated a weighted genetic risk score (GRS) for the relevant exposures using single nucleotide polymorphisms (SNPs) that were independent of each other (*r*^2^ <0.001) and either associated with the exposure at genome-wide significance (*P* < 5 × 10^−8^) or a less stringent cut-off (*P* < 5 × 10^−6^) if there were fewer than 3 SNPs available at *P* < 5 × 10^−8^, in order to mitigate against weak instrument bias. The GRS was calculated by weighting each SNP by the per allele effect on the exposure and then summing the alleles across SNPs. The details are shown in Supplemental Methods. We compared MR results to the confounder-adjusted multivariable regression findings focusing on directional consistency.

## Results

### Discovery ExWAS

Supplemental Table 1 describes the characteristics of the ALSPAC participants. The sample size for infancy, early-, middle-childhood and adolescence was 12761, 12138, 9105, and 7379, the median BMI was 17.2, 16.3, 17.9, and 21.4 kg/m^2^, and the number of exposures was 427, 594, 660 and 842, respectively.

Of the total 2523 exposure-BMI associations explored across all age groups, 172 reached the Bonferroni corrected *P*-value threshold in unadjusted discovery ExWAS analyses, 8 of these were in infancy, 16 in early-childhood, 66 in middle-childhood and 82 in adolescence (Figure 1). The unadjusted associations for all exposures are shown in Figure 2 and Supplemental Table 3. These included previously reported exposure associations, such as maternal BMI during pregnancy, which associated with off-spring BMI in all 4 age groups, and offspring prior BMI, which associated with higher BMI at early-, middle-childhood and adolescence. We also identified several potentially novel risk factors, such as children’s temperament associated with BMI in middle-childhood. For example, child’s shyness and child crying easily at age three were associated with lower BMI in middle-childhood, child being easy-going, higher sociability score, and child preferring to play with others than alone at age three were associated with higher BMI in middle-childhood. Of the 172 associations passing the Bonferroni correction which included 115 exposures (ie, some exposures were associated with BMI at several ages), 3 exposures associated with BMI at all 4 ages, 3 with three, 42 with 2 and 67 with just one of the age groups.

The 172 associations included 8, 16, 66, 82 associations in infancy, early-childhood, middle-childhood and adolescence, respectively, of these, 8, 11, 42, and 26 remained after controlling for confounders (Figure 3). These included previously reported associations such as maternal BMI and offspring prior BMI (Figure 3A–D), and maternal smoking (including smoking pre-pregnancy, during the first three months of pregnancy and the last two weeks of pregnancy) associated with higher BMI at early-, middle-childhood and adolescence (Figure 3B–D). Some potentially novel factors identified in the unadjusted analysis, such as children’s temperament, also remained in the confounder-adjusted analysis. Of the 20 derived variables from the Emotionality Activity Sociability (EAS) scale measured when children were aged three, seven were associated with childhood BMI, including whether children cry easily, whether children are always on the go, shyness (complete cases and prorated), EAS sociability score (complete cases and prorated), and whether child prefer to play with others than alone. For example, children who were described as being always on the go had a 0.16 kg/m^2^ (95% confidence interval (CI) 0.07-0.25) higher BMI compared with those who are not. Children with a mother-reported higher shyness score, those reported to cry easily, and those who scored lower in the sociability score had lower BMI (Figure 3C). Additional adjustment for children’s age and sex were consistent with the confounder adjusted results without children’s age and sex, this additional adjustment had no effect on the selected potentially causal exposures (Supplemental Table 6).

### Replication in independent cohorts

**Figure 4:**
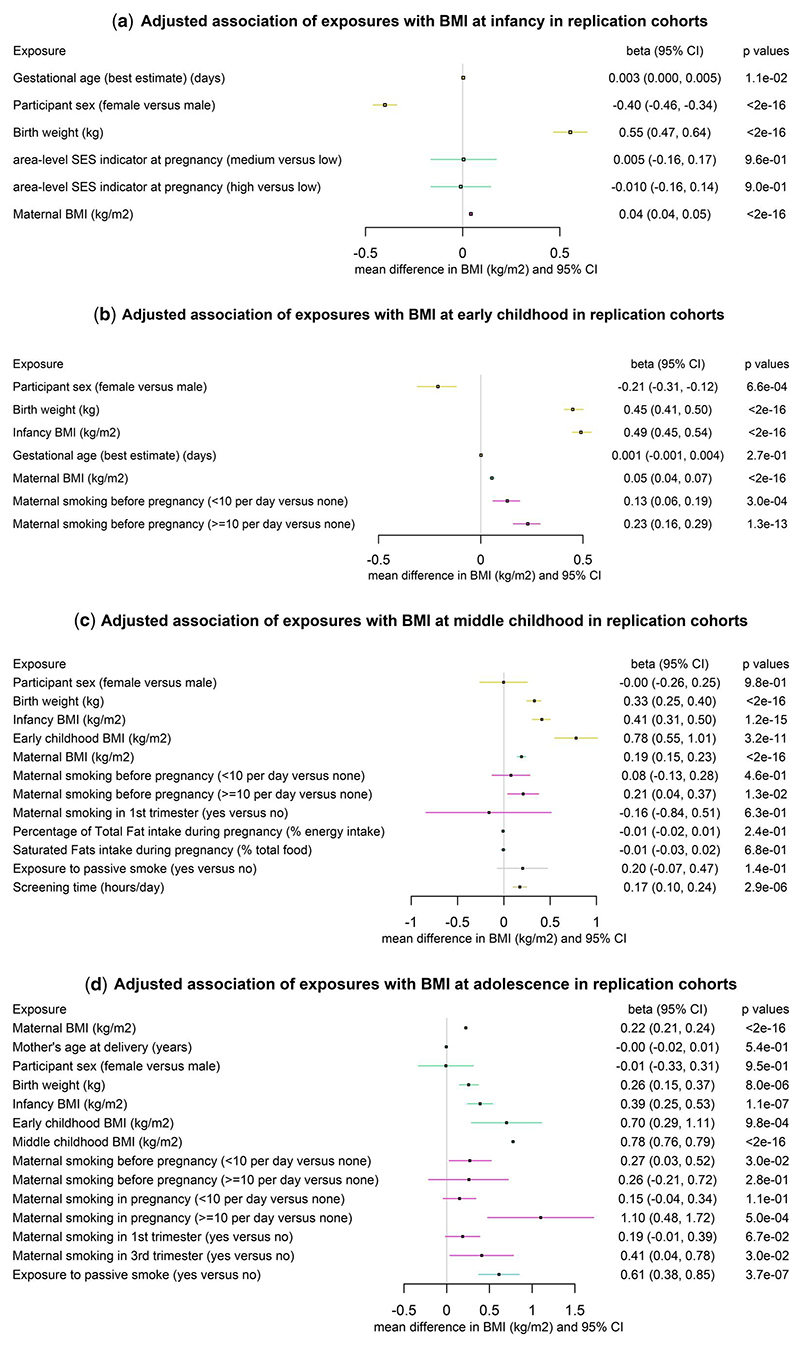
Meta-analysis of replication in other cohorts. The different colors represent different categories of the exposures, the categories are consistent with those shown in Supplemental Table 3.

Supplemental Table 2 describes the characteristics of each of the eight cohorts. The sample size of these cohorts ranges from 1344 (EDEN) to 39835 (DNBC). The pooled sample size for replication ranged from 2631 to 71 056 depending on the specific exposure (Supplemental Table 4). Of the associations that remained after confounder adjustment, it was possible to attempt replication in independent cohort(s) for 5, 6, 11 and 12 exposures in infancy, early-childhood, mid-childhood and adolescence, respectively (Figure 4). We were not able to attempt replication of the other exposures retained in the confounder adjusted analyses. In particular, none of the replication cohorts had measures of the EAS scale meaning that we were unable to explore replication of the temperament and sociability associations. Of these exposures able to attempt replication, 4 (80%), 5 (83%), 7 (63%) and 9 (75%) replicated in infancy, early-childhood, mid-childhood and adolescence, respectively. These included maternal exposures, such as maternal BMI, for which we observed replication at all four ages, maternal smoking in pregnancy with BMI in early-, middle-childhood and adolescence, maternal saturated fat intake during pregnancy with BMI in middle-childhood, and children’s exposures, such as birth weight with BMI at all ages and pre-school TV viewing time with BMI in middle-childhood (Figure 4). For example, BMI in middle-childhood was on average 0.17 kg/m^2^ (95% CI 0.10–0.24) higher per 1 hour/day higher pre-school TV viewing time in the replication pooled samples, compared with 0.21 (0.14, 0.28) in confounder adjusted analyses in ALSPAC. Of the 25 replicating exposure-BMI associations across all age groups, 12 showed low between-cohort heterogeneity (*I*^2^ ≤50%) (Supplemental Figures 1–4), such as maternal BMI, maternal smoking pre-pregnancy, maternal total fat intake and saturated fat intake during pregnancy, birth weight and children’s TV viewing time. For those with large between-cohort heterogeneity, it largely reflected differences in magnitude, with most study results being directionally consistent.

### MR

Our search identified 2 relevant publications^28,30^ (see Supplemental Table 7 for details**)**. Both explored the effect of maternal pregnancy BMI on offspring birth weight, BMI and fat mass and both found causal evidence for an effect of higher maternal pregnancy BMI on higher offspring birth weight, but not subsequent BMI. The largest and most recently published included a total of 9339 infants with numbers varying from 9339 at birth, 8659 at age 1 to 4112 at age 15.

**Figure 5:**
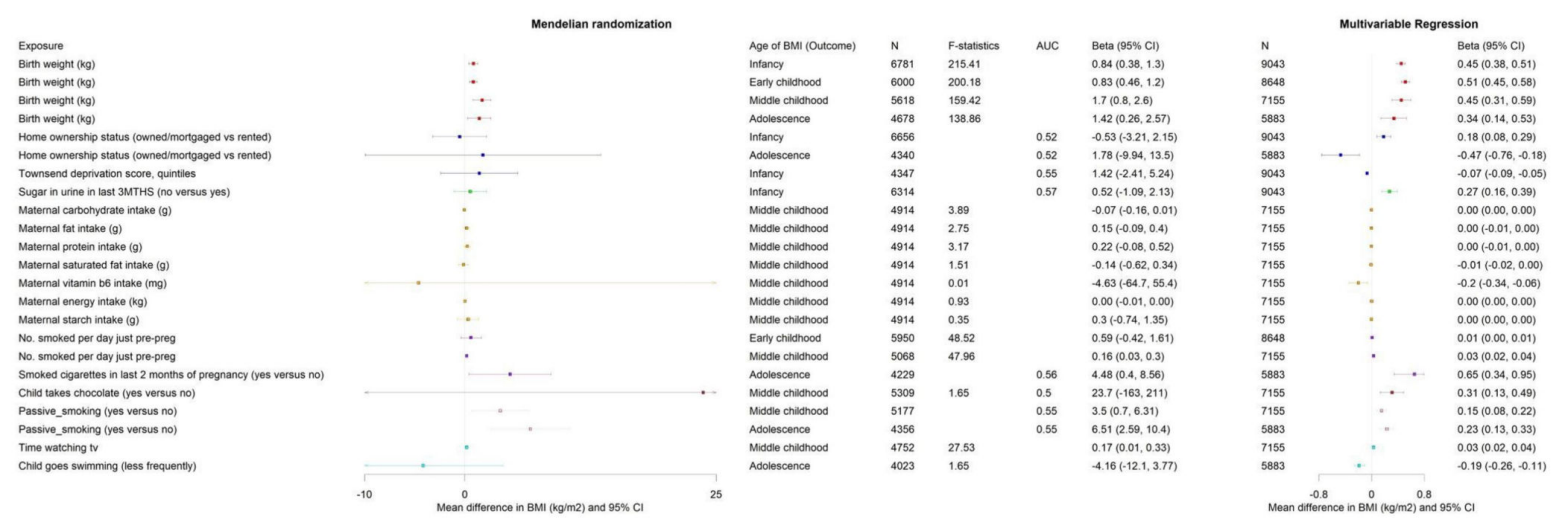
One-sample Mendelian randomization analysis in comparison with multivariable regression in ALSPAC. The different colors represent different categories of the exposures, the categories are consistent with those shown in Supplemental Table 3.

Excluding maternal pregnancy BMI, of the 63 exposures that were confounder-adjusted associated with childhood BMI at any age we identified GWAS that could provide genetic instruments for 16 (Supplemental Table 8) and conducted MR in ALSPAC. The strength of the GRS instruments varied, with F-statistics ranging from 0.01, for maternal pregnancy reported vitamin B6 intake, to 215.4 for birth weight (Figure 5). Sample size ranged from 4023, for the association between physical activity and BMI in adolescence to 6781 for birthweight and BMI in infancy. We found directional consistency in MR results between maternal pregnancy smoking and BMI in adolescence compared with confounder-adjusted results, though with wider confidence intervals in the MR (Figure 5): difference in mean BMI in adolescence comparing any vs no pregnancy smoking: 4.48 (95% CI 0.40-8.56) in MR vs 0.65 (0.34-0.95) in confounder adjusted regression. Similarly, we found directional consistency in MR results between child passive smoking and BMI in middle-childhood and adolescence, as well as TV viewing time and BMI in middle-childhood: 0.17 (95% CI 0.01-0.33) in MR vs 0.03 (95% CI 0.02 to 0.04) in confounder adjusted regression. Sensitivity analyses adjusting for fetal genotype for maternal smoking and TV viewing time were broadly consistent, but gave wider confidence intervals, eg, maternal smoking and BMI in adolescence: 2.33 (95% CI −2.62 to 7.28) in sensitivity analysis vs 4.48 (95% CI 0.40 to 8.56) in main analysis, TV viewing time and BMI in middle-childhood: 0.37 (95% CI −0.10 to 0.84) in sensitivity analysis vs 0.17 (95% CI 0.01-0.33) in main analysis (Supplemental Table 9).

## Discussion

To our knowledge this is the only ExWAS of a wide range of exposures on offspring BMI at different ages across infancy to adolescence. It further adds to previous ExWAS of early life BMI, and ExWAS in general, by seeking external replication and exploring causality with MR. We found robust evidence based on confounder adjustment replication and MR for birth weight with BMI at all ages, and longer early childhood TV viewing time with BMI in middle-childhood. We also found potential effects of some novel exposures, such as children’s temperament and sociability, for which it was not possible to explore replication or undertake MR because these measures are not widely available in studies that also have childhood BMI, confounders and/or relevant genetic data.

Table 1 summarizes of all 64 exposures that remained associated with BMI at any age in the confounder adjusted regression analyses, those that were possible to replicate and undertake MR and the results of the replication/MR with BMI at the relevant ages, for ease of overall interpretation of results and hence potential implications. For example, it can be seen that all of the maternal pregnancy exposures were explored in relation to their impact on BMI at all four age groups, whereas for childhood exposures only age groups occurring after the time of the measurement in childhood could be explored, as we a priori decided we only wanted to explore prospective associations (exposure occurring before outcome). As mother’s report of time spent on watching TV was assessed at age 4, it was only possible to explore its association with BMI in middle-childhood and adolescence. Confounder adjusted associations were found at middle-childhood in ALSPAC, with replication in pooled results from three independent cohorts and having directional support from MR analyses. Thus, this is the exposure with most robust support for a causal effect. Our study also provided supporting evidence regarding current recommendations on restriction of screen time.^31^ More understanding on how TV viewing is associated with higher BMI is needed. It is possible that this association might be confounded by other behaviors, such as less physical activity.^32,33^ Our directionally consistent MR results to some extent argue against this, but having larger studies with genetic data to explore this further would be useful.

Our one-sample MR analysis also supported maternal smoking association with higher childhood BMI in middle childhood and adolescence. That said we acknowledge that the MR analyses were very imprecise and negative control (paternal smoking) did not support a causal effect,^34^ and a novel cross-generational gene by smoking interaction MR analysis suggested no effect of maternal smoking intensity on offspring adult BMI.^35^ The latter study was conducted in the UK Biobank at a different age (40-69 years old) from our study, so the results may not be comparable to our findings. Replication of the one-sample MR in larger samples will be worthwhile.

There is a lack of replication in independent cohorts and/or MR support for the association of higher area deprivation with lower BMI at infancy, maternal BMI with offspring BMI,^28^ and maternal total fat, saturated fat, carbohydrate, protein, vitamin B6, energy, and starch intake during pregnancy, and children’s chocolate intake with higher BMI in middle childhood (Table 1), which suggests that these exposures do not causally affect childhood BMI. For example, in the replication analysis for area deprivation, we found varying associations in different cohorts, eg, GenR and EDEN had different directions of associations, possibly because the two cohorts are only based on one or two cities, with different socioeconomic background compared with other cohorts. The association was also not replicated in other cohorts and not supported by MR (Table 1), supporting the association may not be causal.

The effects of maternal dietary factors are not supported by MR. However, as the genetic instruments for dietary factors have poor strength (*F*-statistics much lower than 10), the MR analysis might be biased by weak instrument. As such, replication using stronger genetic instruments, when available, will be valuable.

We found several exposures for which we were unable to seek replication or perform MR analysis, but are worthwhile to look into in future studies, such as child temperament, sociability and peer problems. Although stress has been shown to be associated with obesity,^36^ the aspects of temperament have been less examined. In our study, seven out of the 20 derived variables from the EAS scale measured when children were aged three, were associated with childhood BMI, including whether children cry easily, whether children are always on the go, shyness (complete cases and prorated), EAS sociability score (complete cases and prorated), and whether child prefer to play with others than alone. One study in Norway showed that children with externalizing temperament had a higher risk to have overweight,^37^ which is partly in line with our findings that children with higher sociability score and preferring to play with others than alone have higher BMI. More studies are needed to examine the reason for our observed association of temperament with higher BMI and to explore whether this might be a causal relation, and if so what the mechanisms for that are. This important question has not been well addressed to date. In addition, some behaviors, such as maternal smoking, may also be a reflection of personality or poor mental health.^38^

In comparison to the three previous ExWAS of childhood BMI^13–15^ (details shown in Supplemental Table 10), the differences in the children’s age at BMI assessment, the exposures covered, whether the studies were cross-sectional or prospective, and the selection of confounders to adjust for between our study and those previous studies makes direct comparisons difficult. For the two previous studies that looked at several different types of risk factor,^14,15^ maternal smoking and birth weight were also found highlighted as associated in one of those two ExWAS.^15^ The other identified risk factors, such as puberty and liver function biomarkers associated with BMI in adolescence in previous ExWAS,^14,15^ were either not retained after confounder adjustment or not measured in ALSPAC. In relation to the previous ExWAS that focused on environmental factors such as features of the built environment and specific pollutants,^13^ children passive smoking was highlighted in that ExWAS as associated with childhood BMI at 6-11 years. It was also a potential risk factor in our analyses in middle-childhood and replicated in MR but not replicated in other cohorts. Notably, despite the large sample size, our study did not identify PM_2.5_ as a risk factor for BMI. The null association between air pollution and BMI has also been shown in other large European cohort studies.^39^

### Strengths, challenges, and limitations

Our study has several strengths. First, it included a relatively large sample size, with a wide range of exposures across childhood. As discussed in a previous ExWAS,^13^ “different exposures may act during different developmental periods, making it important to cover multiple periods”, our study covering different ages filled this research gap and provided valuable information for future interventional studies. Second, we conducted replication analyses in the EU Child Cohort Network, with harmonized methods for data management and analyses.^21^ We performed the replication analyses using DataSHIELD, which is a safe and robust data analysis platform to perform individual-level participant data meta-analyses, without physically transferring data.^21^ Third, we used MR to minimize confounding, which was promoted in a recent commentary on the ExWAS to strengthen causal inference.^16^

Our study also reflected several challenges in using ExWAS to identify modifiable targets. Of the 2523 exposure-BMI associations, only ~7% associations passed Bonferroni-correction, and ~3.4% of the total remained associated after controlling for confounders. Given the hypothesis free nature of ExWAS, we felt it was appropriate to use the conservative Bonferroni-corrected *P*-values to decide which exposure associations to take forward for confounder adjustment. However, this means we could have missed some potentially important effects due to a type 1 error. This is the nature of not attempting to take forward all associations explored in any hypothesis-free study. However, by providing all of these results, Supplemental Table 3 is a useful resource for future research, including by other researchers, that might explore the potential effects of some of the exposures that are close to the Bonferroni-corrected threshold in larger samples. Of those associations that passed the Bonferroni-corrected *P*-value threshold and remained after adjustment for confounders, for more than half (58%) were not able to seek replication in other cohorts, or to assess using MR studies. Again, this is a common challenge of any hypothesis-free study. As noted in the introduction, the tendency for large numbers of studies to repeatedly assess the established exposures, such as physical activity, reflects factors that are commonly measured because they are hypothesized. On the other hand, our hope is that this study will stimulate more studies to assess the potential novel risk factors that we could not explore beyond confounder adjustment.

Beyond these challenges there are some specific limitations. Although we used multivariable regression to control for pre-specified confounders, residual confounding cannot be ruled out as an explanation for some of our potential risk factors, particularly those for which replication and MR could not be undertaken. We used complete case analysis, which is generally unbiased when the chance of having complete data does not depend on the outcome conditional on all covariables in the main analyses.^40^ It is not possible to test this assumption but bias from selection bias due to missing data is less likely where results were replicated as there were differences between the discovery and replication cohorts in amounts of missing data. Most exposures were parental- or child-reported, and these might be misreported. We would expect this to be random and not related to variation in BMI (outcome) which was always assessed after the exposure. Such measurement error would be expected to bias results towards the null in the unadjusted analyses and could bias in either direction in confounder adjusted analyses, depending on the direction and size of association of the error with confounders as well as the outcome.^41^ Due to the limited sample size in ALSPAC, and small number of genetic instruments for some exposures, MR results that we undertook in ALSPAC might be biased towards the confounder adjusted associations due to weak instrument bias for some exposures, such as maternal vitamin B6 intake. It is acknowledged that methods for exposome-wide associations are very varied, and that it covers a variety of very different aims, types of exposures and statistical methods.^42,43^ Some exposure wide-association studies mutually adjust all of the exposures for each other.^42^ That can be useful if the aim is to identify a group of exposures that might accurately predict an outcome. The aim of this study is to identify causal risk factors for variation in childhood BMI. For causal analyses it is important to adjust for prespecified confounders (ie, factors that are known or plausible causes of the exposure and outcome,^44^ as we did, as well as exploring replication and/or MR. When multiple variables are mutually adjusted in a model, the results do not provide unbiased causal estimates as many of the exposures will be adjusted for variables that are not confounders, and some will potentially be on the causal path and we would not want to control for them.^44,45^ It is also likely there will be collider bias and the results from such a model are often not reproducible and difficult to interpret.^45^ Similarly presenting the percent variation of a set of variables does not provide any evidence of causality, is not reproducible and is difficult to interpret.^46^ Thus, for this study we have not mutually adjusted exposures and nor do we present percent variation explained by the exposures.

In conclusion, we found support for the effects of several hypothesized risk factors on BMI, such as maternal higher pregnancy BMI on higher infant BMI, and more offspring TV viewing time in early-childhood on higher subsequent (mid-childhood) BMI, and identified several potential novel risk factors related to childhood BMI, such as child emotion, sociability, and peer problems, which require further exploration. This study highlights the importance of replication and exploring causality in ExWAS.

## Supplementary Material

Supplemental Figures 1-4

Supplemental Methods

## Figures and Tables

**ab:**
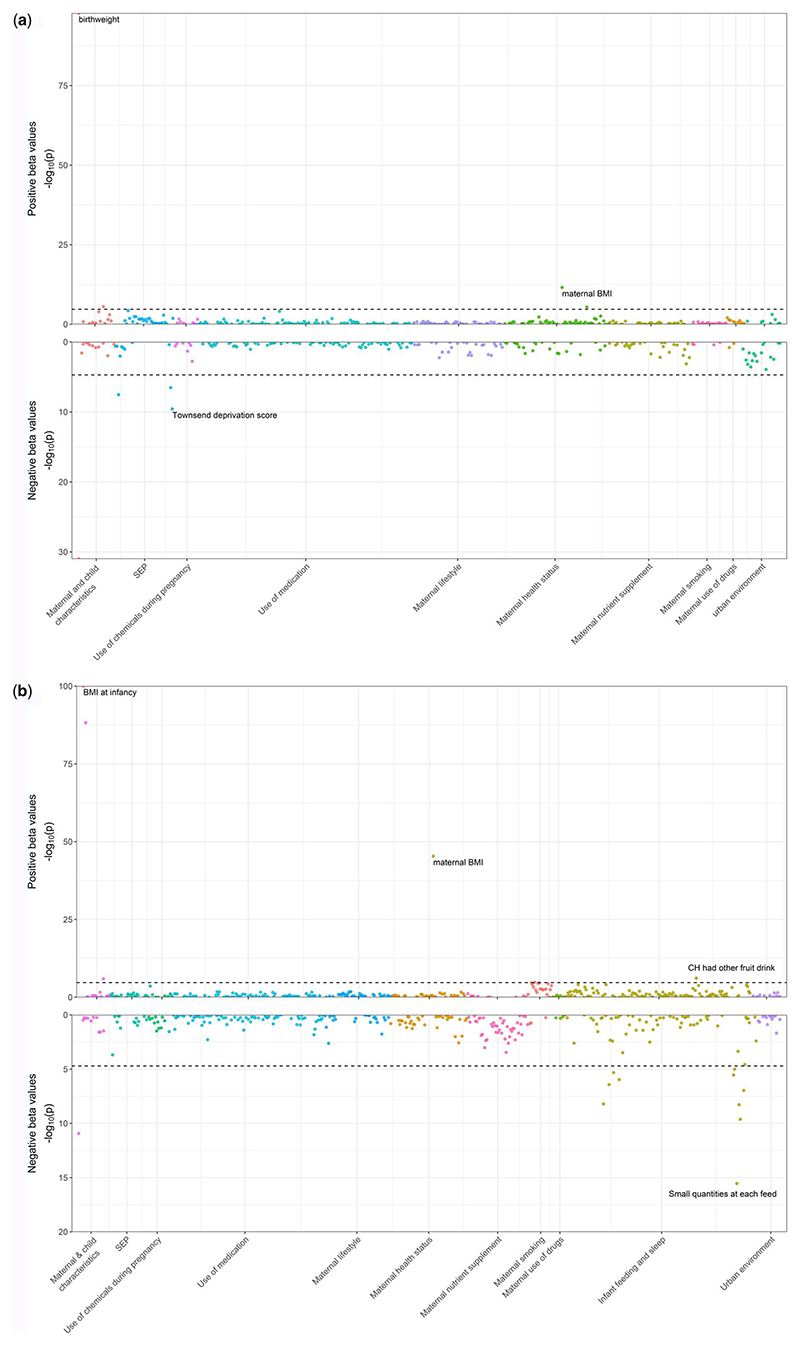


**cd:**
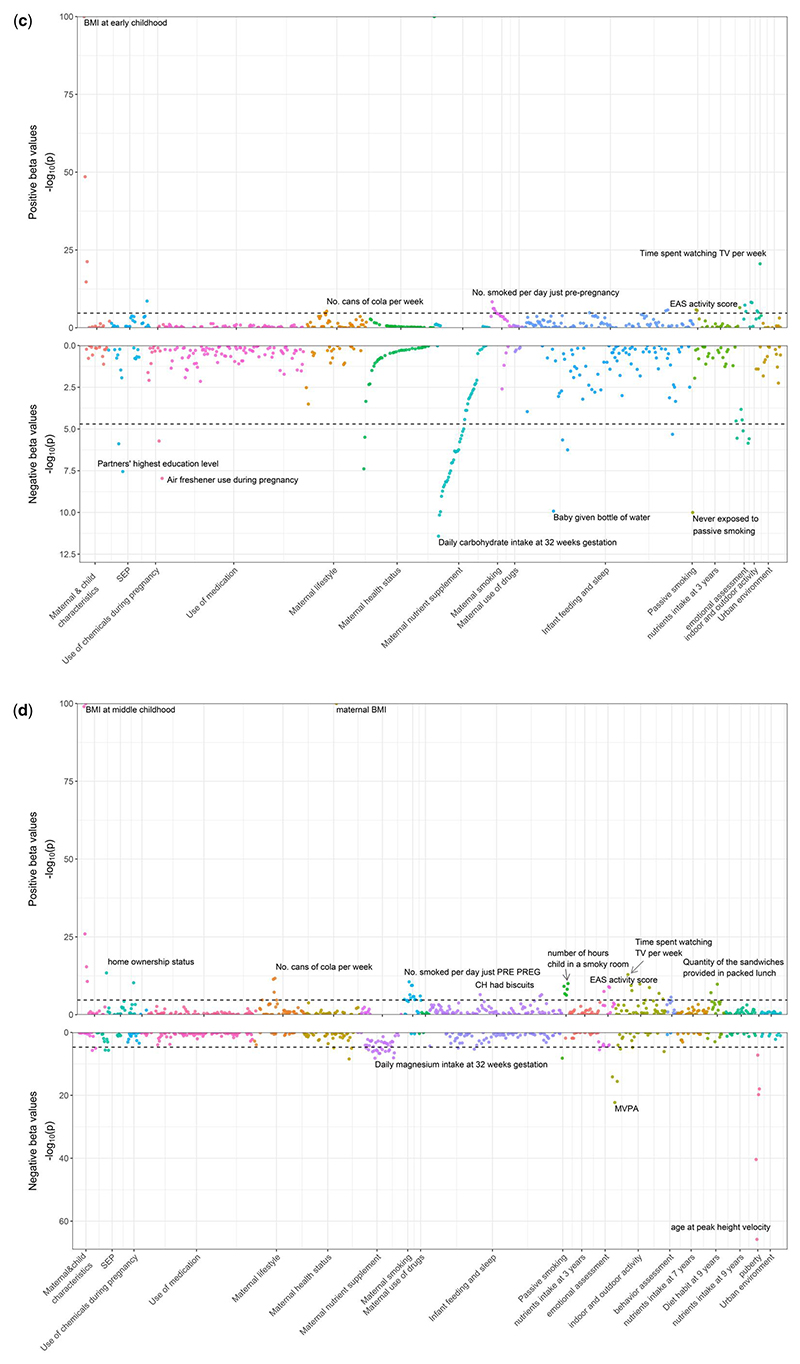


**a:**
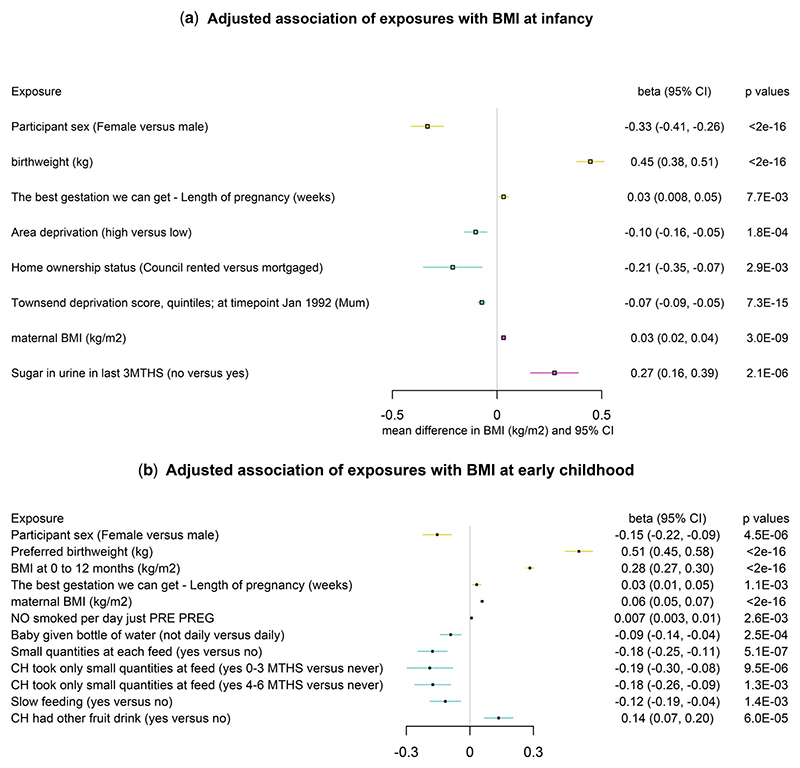


**b:**
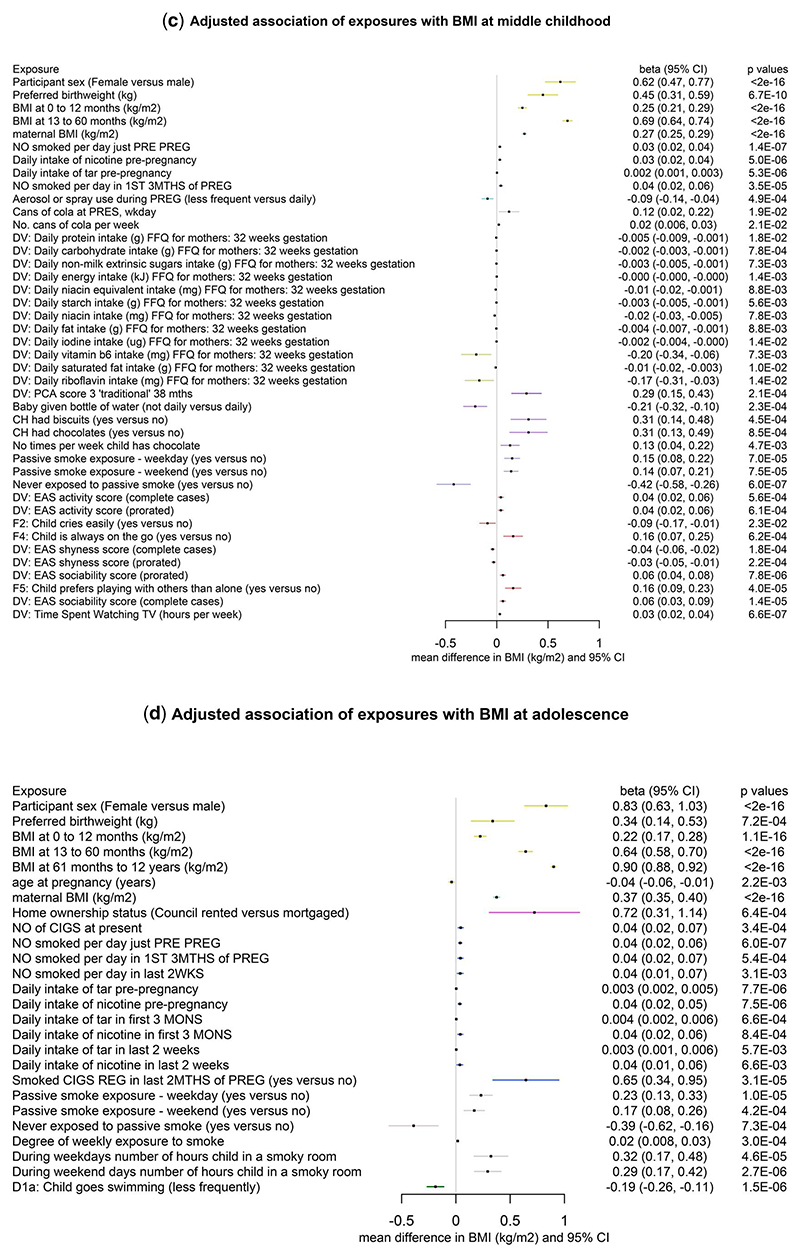


**Table 1:** Summary of findings on replication using multi-cohorts and Mendelian randomization (MR).

Exposure	Age at which associated with BMI after confounder adjustment				Replication	Support from MR
	infancy	early childhood	middle childhood	adolescence		
Participant sex	Y	Y	Y	Y	Y for infancy and early childhood	NP
Preferred birthweight	Y	Y	Y	Y	Y for all ages	Y
BMI at 0 to 12 months	NA	Y	Y	Y	Y for all three ages observed	NP
BMI at 13 to 60 months	NA	NA	Y	Y	Y for the two ages observed	NP
BMI at 61 months to 12 years	NA	NA	NA	Y	Y for adolescence	NP
Area deprivation	Y	N	N	N	N	N
Home ownership status	Y	N	N	Y	NP	N
The best gestation we can get—Length of pregnancy (weeks)	Y	Y	N	N	Y for infancy only	NP
Townsend deprivation score, quintiles; at timepoint January 1992 (Mum)	Y	N	N	N	NP	N
Maternal BMI	Y	Y	Y	Y	Y for all 4 ages	N
Age at pregnancy	N	N	N	Y	N	NP
Sugar in urine in last 3MTHS	Y	N	N	N	NP	N
NO smoked per day just PRE PREG	N	Y	Y	Y	Y for all three ages observed	Y for middle-childhood and adolescence
Daily intake of nicotine pre-pregnancy	N	N	Y	Y	NP	NP
Daily intake of tar pre-pregnancy	N	N	Y	Y	NP	NP
NO smoked per day in first 3 MTHS of PREG	N	N	Y	Y	N	NP
NO of CIGS at present	N	N	N	Y	Y	Y for middle-childhood and adolescence
NO smoked per day in last 2 WKS	N	N	N	Y	NP	NP
Daily intake of tar in first 3 MONS	N	N	N	Y	NP	NP
Daily intake of nicotine in first 3 MONS	N	N	N	Y	NP	NP
Daily intake of tar in last 2 weeks	N	N	N	Y	NP	NP
Daily intake of nicotine in last 2 weeks	N	N	N	Y	NP	NP
Smoked CIGS REG in last 2MTHS of PREG	N	N	N	Y	Y	Y
Passive smoke exposure—weekday	N	N	Y	Y	Y only in adolescence	Y for both middle-childhood and adolescence
Passive smoke exposure—weekend	N	N	Y	Y	Y only in adolescence	Y for both middle-childhood and adolescence
Never exposed to passive smoke	N	N	Y	Y	Y only in adolescence	Y for both middle-childhood and adolescence
Degree of weekly exposure to smoke	N	N	N	Y	NP	NP
During weekdays number of hours child in a smoky room	N	N	N	Y	NP	NP
During weekend days number of hours child in a smoky room	N	N	N	Y	NP	NP
Aerosol or spray use during PREG	N	N	Y	N	NP	NP
Cans of cola at PRES, wkday	N	N	Y	N	NP	NP
No. cans of cola per week	N	N	Y	N	NP	NP
DV: Daily protein in-take (g) FFQ for mothers: 32 weeks gestation	N	N	Y	N	NP	N
DV: Daily carbohydrate intake (g) FFQ for mothers: 32 weeks gestation	N	N	Y	N	NP	N
DV: Daily non-milk extrinsic sugars intake (g) FFQ for mothers: 32 weeks gestation	N	N	Y	N	NP	NP
DV: Daily energy intake (kJ) FFQ for mothers: 32 weeks gestation	N	N	Y	N	NP	N
DV: Daily niacin equivalent intake (mg) FFQ for mothers: 32 weeks gestation	N	N	Y	N	NP	NP
DV: Daily starch intake (g) FFQ for mothers: 32 weeks gestation	N	N	Y	N	NP	N
DV: Daily niacin intake (mg) FFQ for mothers: 32 weeks gestation	N	N	Y	N	NP	NP
DV: Daily fat intake (g) FFQ for mothers: 32 weeks gestation	N	N	Y	N	N	N
DV: Daily iodine intake (μg) FFQ for mothers: 32 weeks gestation	N	N	Y	N	NP	NP
DV: Daily vitamin b6 intake (mg) FFQ for mothers: 32 weeks gestation	N	N	Y	N	NP	N
DV: Daily saturated fat intake (g) FFQ for mothers: 32 weeks gestation	N	N	Y	N	Y	N
DV: Daily riboflavin intake (mg) FFQ for mothers: 32 weeks gestation	N	N	Y	N	NP	NP
DV: PCA score 3 “traditional” 38 mths	N	N	Y	N	NP	NP
Baby given bottle of water	NA	Y	Y	N	NP	NP
Small quantities at each feed	NA	Y	N	N	NP	NP
CH took only small quantities at feed	NA	Y	N	N	NP	NP
Slow feeding	NA	Y	N	N	NP	NP
CH had other fruit drink	NA	Y	N	N	NP	NP
DV: EAS activity score	NA	NA	Y	N	NP	NP
(complete cases) DV: EAS activity score (prorated)	NA	NA	Y	N	NP	NP
CH had biscuits	NA	N	Y	N	NP	NP
CH had chocolates	NA	N	Y	N	NP	N
No times per week child has chocolate	NA	N	Y	N	NP	NP
F2: Child cries easily	NA	NA	Y	N	NP	NP
F4: Child is always on the go	NA	NA	Y	N	NP	NP
DV: EAS shyness score (complete cases)	NA	NA	Y	N	NP	NP
DV: EAS shyness score (prorated)	NA	NA	Y	N	NP	NP
DV: EAS sociability score (prorated)	NA	NA	Y	N	NP	NP
F5: Child prefers playing with others than alone	NA	NA	Y	N	NP	NP
DV: EAS sociability score (complete cases)	NA	NA	Y	N	NP	NP
DV: Time Spent Watching TV per week	NA	NA	Y	N	Y	Y
D1a: Child goes swimming	NA	NA	N	Y	NP	N

N, No; Y, Yes; NA, Not available; NP, not possible currently.

## Data Availability

Data are available by request from the ALSPAC Executive Committee for researchers who meet the criteria for access to confidential data (bristol.ac.uk/alspac/researchers/access/).
