# Supplementary material for: Determinants of body mass index during early life: findings from an exposome-wide association study with follow-up replication and Mendelian randomization analyses
Source: Exposome. Author manuscript; Available in PMC 2026 Jun 24. (PMC7619207; doi:10.1093/exposome/osaf004)

Supplemental Figure 1. The association of each exposure with BMI at infancy in replication cohorts

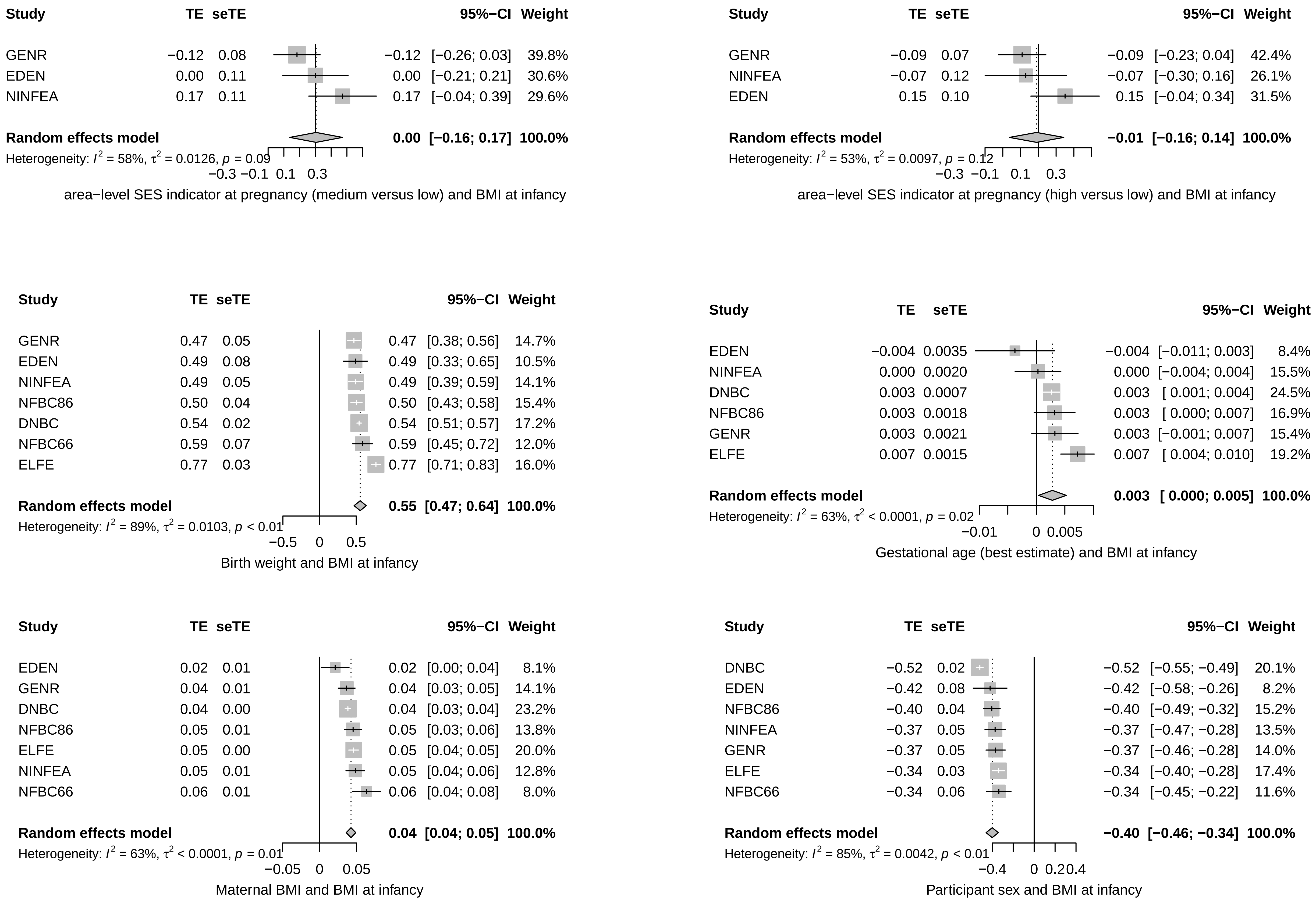

Supplemental Figure 2. The association of each exposure with BMI at early childhood in replication cohorts

Birth weight and early childhood BMI

Study	TE	seTE	95%−CI	Weight
EDEN	0.32	0.08	0.32 [0.17; 0.47]	7.0%
NINFEA	0.39	0.05	0.39 [0.29; 0.49]	11.8%
GENR	0.41	0.04	0.41 [0.33; 0.50]	13.9%
NFBC86	0.44	0.04	0.44 [0.37; 0.51]	16.5%
NFBC66	0.47	0.07	0.47 [0.34; 0.60]	8.6%
DNBC	0.50	0.02	0.50 [0.46; 0.54]	23.4%
ELFE	0.53	0.03	0.53 [0.47; 0.59]	18.7%
**Random effects model**			**0.46 [0.41; 0.50]**	**100.0%**

Heterogeneity: $I^2 = 56\%$, $\tau^2 = 0.0019$, $p = 0.03$

infancy BMI and early childhood BMI

Study	TE	seTE	95%−CI	Weight
NINFEA	0.38	0.01	0.38 [0.36; 0.41]	14.3%
EDEN	0.44	0.02	0.44 [0.40; 0.49]	13.1%
ELFE	0.48	0.01	0.48 [0.46; 0.50]	14.8%
NFBC66	0.51	0.02	0.51 [0.48; 0.54]	14.1%
NFBC86	0.53	0.01	0.53 [0.51; 0.55]	14.5%
DNBC	0.55	0.01	0.55 [0.54; 0.56]	15.0%
GENR	0.55	0.01	0.55 [0.52; 0.58]	14.2%
**Random effects model**			**0.49 [0.45; 0.54]**	**100.0%**

Heterogeneity: $I^2 = 97\%$, $\tau^2 = 0.0033$, $p < 0.01$

Gestational age (best estimate) and early childhood BMI

Study	TE	seTE	95%−CI	Weight
EDEN	−0.005	0.0031	−0.005 [−0.011; 0.002]	9.3%
ELFE	−0.001	0.0015	−0.001 [−0.004; 0.002]	19.3%
NINFEA	0.000	0.0021	0.000 [−0.004; 0.004]	14.9%
DNBC	0.003	0.0009	0.003 [0.001; 0.004]	23.7%
GENR	0.003	0.0020	0.003 [−0.001; 0.007]	15.3%
NFBC86	0.005	0.0017	0.005 [0.002; 0.008]	17.5%
**Random effects model**			**0.001 [−0.001; 0.004]**	**100.0%**

Heterogeneity: $I^2 = 65\%$, $\tau^2 < 0.0001$, $p = 0.01$

Maternal BMI and early childhood BMI

Study	TE	seTE	95%−CI	Weight
ELFE	0.04	0.00	0.04 [0.03; 0.04]	15.9%
DNBC	0.04	0.00	0.04 [0.04; 0.05]	16.2%
EDEN	0.04	0.01	0.04 [0.03; 0.06]	12.5%
GENR	0.05	0.01	0.05 [0.04; 0.06]	14.5%
NINFEA	0.06	0.01	0.06 [0.05; 0.08]	14.0%
NFBC66	0.07	0.01	0.07 [0.06; 0.09]	12.2%
NFBC86	0.08	0.01	0.08 [0.07; 0.09]	14.7%
**Random effects model**			**0.05 [0.04; 0.07]**	**100.0%**

Heterogeneity: $I^2 = 91\%$, $\tau^2 = 0.0002$, $p < 0.01$

Maternal smoking before pregnancy (<10 per day versus none) and early childhood BMI

Study	TE	seTE	95%−CI	Weight
GENR	0.01	0.06	0.01 [−0.11; 0.12]	19.6%
NFBC86	0.11	0.07	0.11 [−0.02; 0.25]	15.3%
NINFEA	0.14	0.09	0.14 [−0.04; 0.31]	10.0%
NFBC66	0.16	0.07	0.16 [0.02; 0.29]	15.5%
ELFE	0.17	0.04	0.17 [0.09; 0.26]	32.1%
EDEN	0.22	0.11	0.22 [0.01; 0.43]	7.4%
**Random effects model**			**0.13 [0.07; 0.19]**	**100.0%**

Heterogeneity: $I^2 = 20\%$, $\tau^2 = 0.0011$, $p = 0.28$

Maternal smoking before pregnancy (>=10 per day versus none) and early childhood BMI

Study	TE	seTE	95%−CI	Weight
GENR	0.12	0.07	0.12 [−0.01; 0.25]	16.6%
NINFEA	0.18	0.09	0.18 [−0.01; 0.36]	9.3%
NFBC86	0.20	0.05	0.20 [0.10; 0.30]	23.9%
ELFE	0.28	0.04	0.28 [0.20; 0.35]	36.5%
EDEN	0.29	0.09	0.29 [0.11; 0.47]	9.9%
NFBC66	0.38	0.15	0.38 [0.08; 0.68]	3.8%
**Random effects model**			**0.23 [0.17; 0.29]**	**100.0%**

Heterogeneity: $I^2 = 22\%$, $\tau^2 = 0.0012$, $p = 0.27$

Participant sex and early childhood BMI

Study	TE	seTE	95%−CI	Weight
DNBC	−0.45	0.02	−0.45 [−0.49; −0.41]	15.3%
ELFE	−0.26	0.03	−0.26 [−0.32; −0.20]	15.1%
EDEN	−0.19	0.07	−0.19 [−0.33; −0.04]	12.7%
NINFEA	−0.16	0.05	−0.16 [−0.26; −0.06]	14.1%
NFBC66	−0.14	0.06	−0.14 [−0.25; −0.03]	13.8%
GENR	−0.13	0.05	−0.13 [−0.22; −0.04]	14.3%
NFBC86	−0.12	0.04	−0.12 [−0.19; −0.04]	14.7%
**Random effects model**			**−0.21 [−0.33; −0.09]**	**100.0%**

Heterogeneity: $I^2 = 94\%$, $\tau^2 = 0.0242$, $p < 0.01$

Supplemental Figure 3. The association of each exposure with BMI at middle childhood in replication cohorts

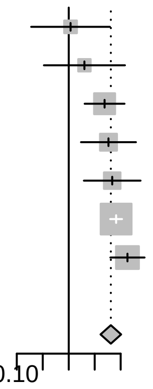

Study	TE	seTE	95%−CI	Weight
EDEN	0.01	0.15	0.01 [−0.29; 0.32]	5.8%
HGS	0.12	0.16	0.12 [−0.19; 0.43]	5.5%
GENR	0.28	0.08	0.28 [0.12; 0.43]	15.8%
NFBC66	0.31	0.11	0.31 [0.09; 0.52]	10.3%
NINFEA	0.33	0.11	0.33 [0.12; 0.55]	9.8%
DNBC	0.37	0.02	0.37 [0.32; 0.41]	34.1%
NFBC86	0.45	0.07	0.45 [0.32; 0.58]	18.7%
**Random effects model**			**0.32 [0.24; 0.40]**	**100.0%**

Heterogeneity: $I^2 = 43\%$, $\tau^2 = 0.0043$, $p = 0.10$

Birth weight and middle childhood BMI

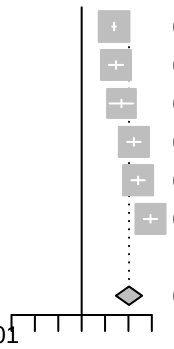

Study	TE	seTE	95%−CI	Weight
DNBC	0.277	0.0084	0.277 [0.261; 0.294]	17.5%
NINFEA	0.294	0.0307	0.294 [0.234; 0.354]	16.7%
EDEN	0.340	0.0515	0.340 [0.239; 0.441]	15.4%
GENR	0.446	0.0292	0.446 [0.389; 0.504]	16.8%
NFBC66	0.483	0.0293	0.483 [0.425; 0.540]	16.8%
NFBC86	0.589	0.0294	0.589 [0.531; 0.646]	16.8%
**Random effects model**			**0.405 [0.293; 0.517]**	**100.0%**

Heterogeneity: $I^2 = 97\%$, $\tau^2 = 0.0186$, $p < 0.01$

Infancy BMI and middle childhood BMI

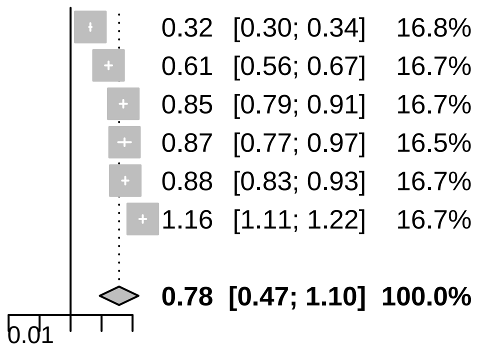

Study	TE	seTE	95%−CI	Weight
DNBC	0.32	0.01	0.32 [0.30; 0.34]	16.8%
NINFEA	0.61	0.03	0.61 [0.56; 0.67]	16.7%
NFBC66	0.85	0.03	0.85 [0.79; 0.91]	16.7%
EDEN	0.87	0.05	0.87 [0.77; 0.97]	16.5%
GENR	0.88	0.03	0.88 [0.83; 0.93]	16.7%
NFBC86	1.16	0.03	1.16 [1.11; 1.22]	16.7%
**Random effects model**			**0.78 [0.47; 1.10]**	**100.0%**

Heterogeneity: $I^2 = 100\%$, $\tau^2 = 0.1516$, $p < 0.01$

Early childhood BMI and middle childhood BMI

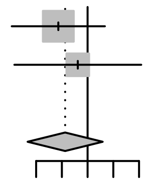

Study	TE	seTE	95%−CI	Weight
GENR	−0.01	0.01	−0.01 [−0.03; 0.01]	65.0%
EDEN	−0.00	0.01	−0.00 [−0.03; 0.02]	35.0%
**Random effects model**			**−0.01 [−0.02; 0.01]**	**100.0%**

Heterogeneity: $I^2 = 0\%$, $\tau^2 = 0$, $p = 0.63$

Percentage of Total Fat intake during pregnancy and middle childhood BMI

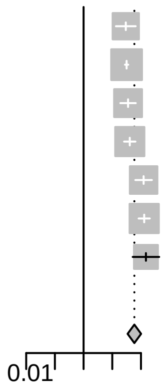

Study	TE	seTE	95%−CI	Weight
EDEN	0.15	0.02	0.15 [0.11; 0.18]	12.7%
DNBC	0.15	0.00	0.15 [0.14; 0.16]	17.4%
NINFEA	0.15	0.01	0.15 [0.13; 0.18]	14.3%
GENR	0.16	0.01	0.16 [0.14; 0.18]	15.6%
NFBC66	0.21	0.01	0.21 [0.18; 0.24]	13.9%
NFBC86	0.21	0.01	0.21 [0.19; 0.23]	15.7%
HGS	0.22	0.02	0.22 [0.17; 0.26]	10.5%
**Random effects model**			**0.18 [0.15; 0.20]**	**100.0%**

Heterogeneity: $I^2 = 89\%$, $\tau^2 = 0.0008$, $p < 0.01$

Maternal BMI and middle childhood BMI

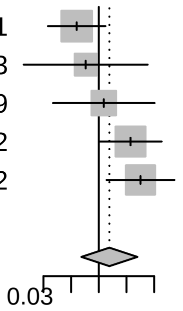

Study	TE	seTE	95%−CI	Weight
GENR	−0.16	0.11	−0.16 [−0.37; 0.05]	24.9%
EDEN	−0.09	0.23	−0.09 [−0.54; 0.35]	12.7%
NINFEA	0.04	0.19	0.04 [−0.33; 0.40]	16.0%
NFBC66	0.23	0.12	0.23 [0.00; 0.45]	23.8%
NFBC86	0.30	0.12	0.30 [0.06; 0.55]	22.6%
**Random effects model**			**0.08 [−0.13; 0.28]**	**100.0%**

Heterogeneity: $I^2 = 62\%$, $\tau^2 = 0.0316$, $p = 0.03$

Maternal smoking before pregnancy (<10 per day versus none) and middle childhood BMI

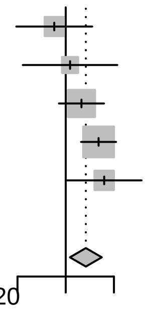

Study	TE	seTE	95%−CI	Weight
NINFEA	−0.12	0.20	−0.12 [−0.51; 0.28]	13.7%
NFBC66	0.05	0.25	0.05 [−0.45; 0.54]	9.6%
GENR	0.16	0.12	0.16 [−0.07; 0.40]	27.5%
NFBC86	0.34	0.09	0.34 [0.16; 0.52]	35.2%
EDEN	0.40	0.20	0.40 [0.01; 0.79]	14.0%
**Random effects model**			**0.21 [0.04; 0.37]**	**100.0%**

Heterogeneity: $I^2 = 34\%$, $\tau^2 = 0.0116$, $p = 0.20$

Maternal smoking before pregnancy (>=10 per day versus none) and middle childhood BMI

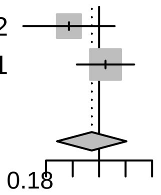

Study	TE	seTE	95%−CI	Weight
EDEN	−0.02	0.02	−0.02 [−0.06; 0.01]	37.6%
GENR	0.00	0.01	0.00 [−0.02; 0.03]	62.4%
**Random effects model**			**−0.01 [−0.03; 0.02]**	**100.0%**

Heterogeneity: $I^2 = 43\%$, $\tau^2 = 0.0002$, $p = 0.18$

Saturated Fats intake during pregnancy and middle childhood BMI

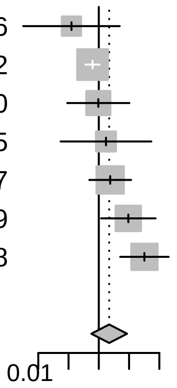

Study	TE	seTE	95%−CI	Weight
HGS	−0.18	0.16	−0.18 [−0.50; 0.14]	8.5%
DNBC	−0.04	0.02	−0.04 [−0.09; 0.01]	21.5%
NINFEA	−0.00	0.10	−0.00 [−0.21; 0.20]	13.3%
EDEN	0.05	0.15	0.05 [−0.25; 0.35]	9.1%
NFBC86	0.08	0.07	0.08 [−0.06; 0.21]	17.1%
NFBC66	0.19	0.09	0.19 [0.01; 0.38]	14.7%
GENR	0.30	0.08	0.30 [0.14; 0.46]	15.8%
**Random effects model**			**0.07 [−0.05; 0.19]**	**100.0%**

Heterogeneity: $I^2 = 74\%$, $\tau^2 = 0.0163$, $p < 0.01$

Participant sex and middle childhood BMI

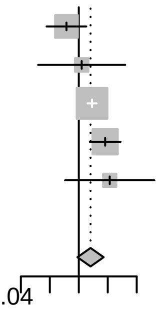

Study	TE	seTE	95%−CI	Weight
EDEN	−0.21	0.17	−0.21 [−0.56; 0.13]	21.0%
HGS	0.05	0.38	0.05 [−0.70; 0.80]	7.4%
DNBC	0.23	0.04	0.23 [0.15; 0.31]	38.5%
GENR	0.45	0.14	0.45 [0.19; 0.72]	26.0%
NINFEA	0.53	0.39	0.53 [−0.24; 1.30]	7.1%
**Random effects model**			**0.20 [−0.02; 0.43]**	**100.0%**

Heterogeneity: $I^2 = 60\%$, $\tau^2 = 0.0331$, $p = 0.04$

Exposure to passive smoke and middle childhood BMI

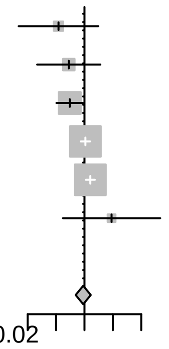

Study	TE	seTE	95%−CI	Weight
EDEN	−1.83	1.43	−1.83 [−4.64; 0.98]	3.3%
NINFEA	−1.10	1.14	−1.10 [−3.33; 1.12]	5.0%
GENR	−1.04	0.48	−1.04 [−1.97; −0.10]	18.1%
DNBC	0.07	0.17	0.07 [−0.26; 0.39]	35.5%
NFBC86	0.41	0.16	0.41 [0.09; 0.73]	35.8%
NFBC66	1.91	1.75	1.91 [−1.52; 5.34]	2.3%
**Random effects model**			**−0.09 [−0.62; 0.44]**	**100.0%**

Heterogeneity: $I^2 = 62\%$, $\tau^2 = 0.1800$, $p = 0.02$

Smoking in 1st trimester and middle childhood BMI

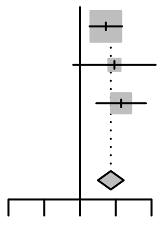

Study	TE	seTE	95%−CI	Weight
HGS	0.14	0.05	0.14 [0.05; 0.24]	63.2%
EDEN	0.19	0.12	0.19 [−0.04; 0.42]	9.8%
GENR	0.23	0.07	0.23 [0.09; 0.37]	26.9%
**Random effects model**			**0.17 [0.10; 0.24]**	**100.0%**

Heterogeneity: $I^2 = 0\%$, $\tau^2 = 0$, $p = 0.59$

Screening time and middle childhood BMI

Supplemental Figure 4. The association of each exposure with BMI at adolescence in replication cohorts

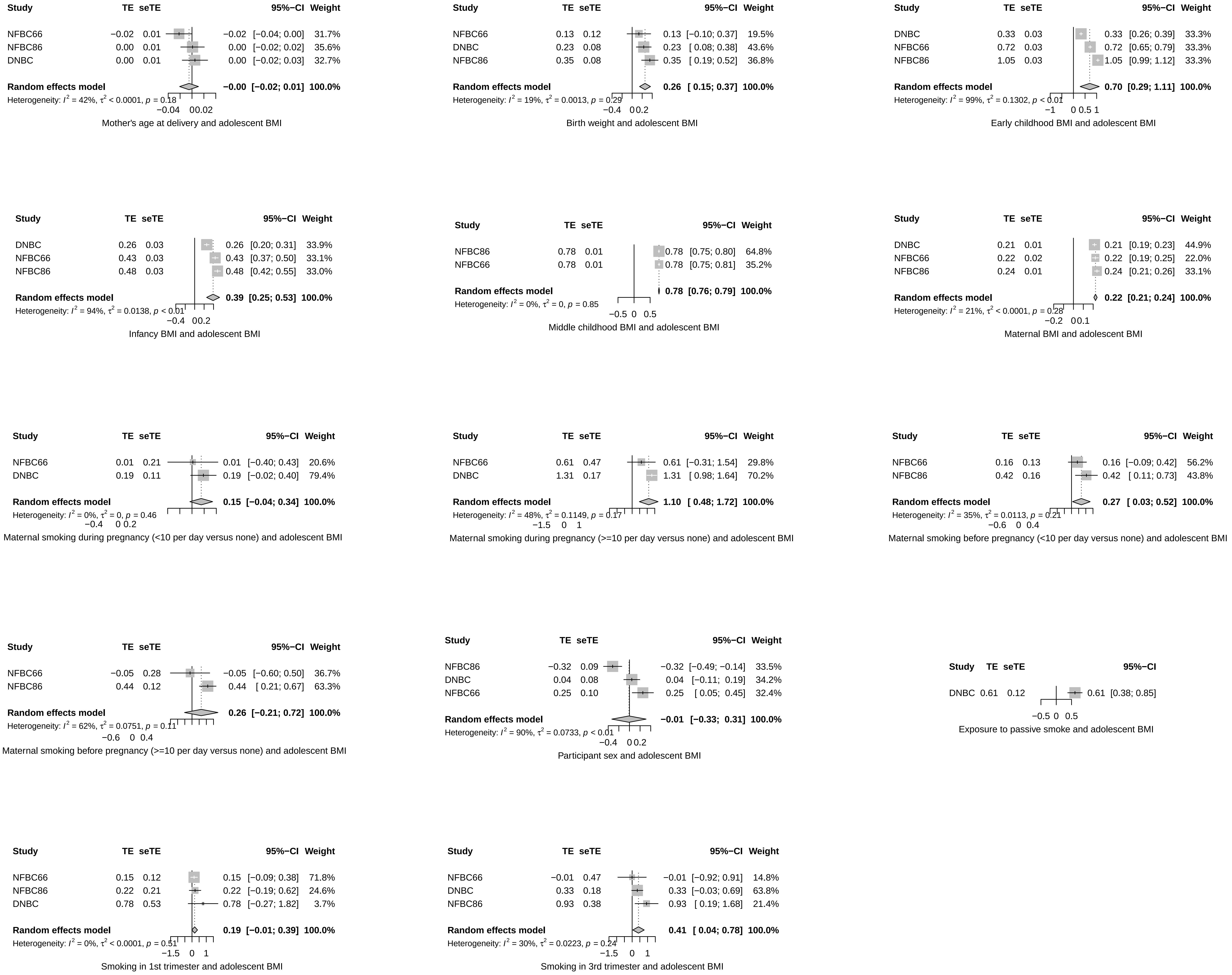

Supplement: Supplemental Figures 1-4 [file EMS212766-supplement-Supplemental_Figures_1_4.pdf]
