# Supplementary material for: Determinants of body mass index during early life: findings from an exposome-wide association study with follow-up replication and Mendelian randomization analyses
Source: Exposome. Author manuscript; Available in PMC 2026 Jun 24. (PMC7619207; doi:10.1093/exposome/osaf004)
Supplement: Supplemental Methods [file EMS212766-supplement-Supplemental_Methods.docx]

**Supplemental Methods**

1. **Description of confounder assessment in ALSPAC**

Maternal age, education, parity, BMI, smoking and ethnicity and childhood prior BMI were potential confounders. Maternal age was obtained from birth records or pregnancy questionnaires. Maternal education, parity, weight, height, smoking and ethnicity were obtained during pregnancy using questionnaires. Maternal education was categorized to CSE, Vocational, O level, A level and Degree. Maternal smoking was assessed by asking mothers whether they have smoked during pregnancy, and categorized to yes and no.

1. **Description of the included cohorts**

The NINFEA cohort is an Italian birth cohort started in 2005 in the city of Turin and then extended to the rest of Italy.^1^ Recruitment was carried out from 2005 to 2016 via Internet and the media. Members of the cohorts are children born to mothers who have access to the internet, have enough knowledge of Italian language to complete online questionnaires and volunteer to participate any time during the pregnancy completing the first baseline questionnaire on general health and exposures before and during pregnancy. The Ethical Committee of the San Giovanni Battista Hospital and CTO/CRF/Maria Adelaide Hospital of Turin approved the NINFEA study and all the participants gave informed consent at enrolment. In this study, we used data from up to 4878 children in the analysis.

DNBC is the Danish national birth cohort, comprising approximately 100,000 mothers and their children. Mothers were recruited by their general practitioners during early gestation, from 1996 through 2002. It has followed them and their children since then, largely through record linkage.^2^ The Committee for Biomedical Research Ethics in Denmark approved the collection of data in the DNBC ((KF)01–471/94). Written informed consent was obtained from mothers upon recruitment, covering both mothers’ participation and children’s participation in the DNBC until they turn 18 years of age. In this study we used data from up to 39,835 children in the analysis.

GenR is a population-based prospective cohort study from fetal life until adulthood, conducted in Rotterdam, the second largest city in the Netherlands.^3^ In total, 9778 mothers with a delivery date from April 2002 until January 2006 were enrolled in the study. Response at baseline was 61%. The GenR study has been approved by the Medical Ethical Committee of the Erasmus MC, University Medical Center in Rotterdam. Written informed consent was obtained from the parents or legal representatives of the children. In this study we used data from up to 3,349 children in the analysis.

ELFE is a French national longitudinal birth cohort with more than 18,000 children included at birth.^4^ Recruitment took place on 25 selected days during 4 periods in 2011. The inclusion criteria were birth at 33 weeks’ amenorrhea or more, singleton or twin birth, and mother > 18 years, who gave informed consent and did not plan to leave metropolitan France within 3 years. Among eligible mothers, 51% agreed to participate (N = 18,040). The ELFE study was approved by an ethics committee (Comité de Protection des Personnes), the national committee on information concerning health research (Comité Consultatif sur le Traitement de l’Information en Matière de Recherche dans le domaine de la Santé), and the data protection authority (Commission Nationale de l’Informatique et des Libertés). In this study we used data from up to 14,335 children in the analysis.

EDEN is a prospective French cohort.^5^ It recruited 2002 pregnant women (before 24 weeks' amenorrhea) older than 18 years old between 2003 and 2006 in two university hospital centres in Nancy and Poitiers. Exclusion criteria were multiple pregnancies, known diabetes before pregnancy, French illiteracy or planning to move out of the region within the next 3 years. 53% of mothers were eligible. Both parents gave their written consent when their child was included. The Data Protection Authority and the relevant ethical research committee of Kremlin‐Bicêtre Hospital gave their approval for the study. In this study we used data from up to 1344 children in the analysis.

NFBC66 and NFBC86 are two longitudinal birth cohorts established to study factors affecting preterm birth and consequent morbidity in the two northernmost provinces of Finland, Oulu and Lapland.^6,7^ The NFBC66 includes 12,058 live births (12,231 children) covering 96% of all eligible births in this region during January-December 1966. Two decades later, a second cohort of 9,432 births (9,479 children) was obtained (NFBC86) which covered 99% of all the deliveries taking place in the target regions during July 1985-June 1986. In both cohorts, mothers and children have been followed-up since mothers enrolled at their first antenatal clinic visit (10-16th week). For NFBC86, the 16-year follow-up data collection (2001-2002) included clinical examination and serum collection for 6,621 adolescents (71% of the original cohort). Ethical approval for the NFBC86 and NFBC66 study was obtained from the Ethics Committee of Northern Ostrobothnia Hospital District, Finland. NFBC86 received ethical approval from Ethics Committee of Northern Ostrobothnia Hospital District and Oulu University, Faculty of Medicine, Oulu, Finland. In this study we used data from up to 2831 children from NFBC66 and 6482 from NFBC86 in the analysis.

HGS was a large-scale cross-sectional study in Greece, initiated in May 2007 and completed in June 2009.^8^ The population under study comprised of schoolchildren aged 9-13 years attending the fifth and sixth grades from primary schools located in municipalities within the counties of Attica, Aitoloakarnania, Thessaloniki and Iraklio. Approval to conduct the study was granted by the Greek Ministry of National Education and the Ethical Committee of Harokopio University of Athens (Reference code: 16/19-12-2006). In this study we used data from up to 2172 children in the analysis.

1. **Description of genotyping and quality control in ALSPAC**

ALSPAC children were genotyped using Illumina HumanHap550 quad, and ALSPAC mothers were genotyped using Illumina human660K quad. Genotype data for both ALSPAC mothers and children were imputed against the Haplotype Reference Consortium v1.1 reference panel (HRC.r1.1) using the Michigan imputation server after performing the quality control (QC) procedure. The QC procedure comprised: minor allele frequency (MAF) ≥1%, a call rate ≥95%, in Hardy-Weinberg equilibrium (HWE), correct sex assignment, no evidence of cryptic relatedness, and of European descent.

**4. Details of Mendelian randomization analysis in ALSPAC**

PLINK (v1.9) was used to construct the GRS for each exposure. All GRS were transformed to standard deviation units. We used the two-stage least-squares (2SLS),[^30^](#_ENREF_30) to obtain the MR effect estimate, with adjustment for the first 10 genetic principal components (PCs), age at BMI assessment and sex. In these 2SLS analyses we made sure that the same units and categories of exposure were used as in the adjusted multivariable regression analyses, so that the two are estimating the same association and effect estimates can be compared.

To assess the relevance of the GRS to each exposure, we undertook linear (for continuous exposures), logistic (for binary exposures) and ordinal logistic (for ordered categorical exposures) regressions of the associations of the GRS with the exposure in the appropriate sample population (e.g., for a GRS of maternal BMI we explored its association in ALSPAC mothers). Instrument strength was assessed with F-statistics (for continuous exposures), and by using the area under the receiver operating characteristic (ROC) curve and pseudo R^2^ (for binary exposures and ordered categorical exposures). The adjustment for PCs and restriction of MR to European ancestry participants was done to mitigate bias due to confounding between the GRS and outcome (childhood BMI), which can occur as a result of population stratification. To explore bias due to violation of the MR exclusion restriction criteria and via offspring genotype, we adjusted for offspring genotype in the maternal exposure GRS-BMI associations. We adjusted for maternal genotype in the offspring exposure GRS-BMI associations to control for maternal genetic confounding of the GRS-outcome association.

1. Richiardi L, Baussano I, Vizzini L, et al. Feasibility of recruiting a birth cohort through the Internet: the experience of the NINFEA cohort. *European journal of epidemiology* 2007; **22**(12): 831-7.

2. Olsen J, Melbye M, Olsen SF, et al. The Danish National Birth Cohort--its background, structure and aim. *Scand J Public Health* 2001; **29**(4): 300-7.

3. Kooijman MN, Kruithof CJ, van Duijn CM, et al. The Generation R Study: design and cohort update 2017. *European journal of epidemiology* 2016; **31**(12): 1243-64.

4. Charles MA, Thierry X, Lanoe JL, et al. Cohort Profile: The French national cohort of children (ELFE): birth to 5 years. *International journal of epidemiology* 2020; **49**(2): 368-9j.

5. Heude B, Forhan A, Slama R, et al. Cohort Profile: The EDEN mother-child cohort on the prenatal and early postnatal determinants of child health and development. *International journal of epidemiology* 2016; **45**(2): 353-63.

6. Rantakallio P. The longitudinal study of the northern Finland birth cohort of 1966. *Paediatr Perinat Epidemiol* 1988; **2**(1): 59-88.

7. Santos Ferreira DL, Williams DM, Kangas AJ, et al. Association of pre-pregnancy body mass index with offspring metabolic profile: Analyses of 3 European prospective birth cohorts. *PLoS medicine* 2017; **14**(8): e1002376.

8. Moschonis G, Kalliora AC, Costarelli V, et al. Identification of lifestyle patterns associated with obesity and fat mass in children: the Healthy Growth Study. *Public Health Nutr* 2014; **17**(3): 614-24.
